# A novel mutation of *WFS1* gene leading to increase ER stress and cell apoptosis is associated an autosomal dominant form of Wolfram syndrome type 1

**DOI:** 10.1186/s12902-021-00748-z

**Published:** 2021-04-21

**Authors:** Yingying Gong, Li Xiong, Xiujun Li, Lei Su, Haipeng Xiao

**Affiliations:** 1 Department of Endocrinology, The First Affiliated Hospital of Sun Yat-sen University, 58 Zhongshan Road 2, Guangzhou, 510080 P. R. China; 2 Department of Geriatrics, The First Affiliated Hospital of Sun Yat-sen University, Guangzhou, 510080 Guangdong China

**Keywords:** Wolfram syndrome, *WFS1* gene, Mutation, ER stress

## Abstract

**Background:**

Wolfram syndrome (WS) is a rare autosomal recessive disorder characterized by diabetes insipidus, diabetes mellitus, optic atrophy and deafness. Mutations in Wolfram syndrome 1 (*WFS1*) gene may cause dysregulated endoplasmic reticulum (ER)-stress and cell apoptosis, contributing to WS symptoms. The aim of this study was to identify the molecular etiology of a case of WS and to explore the functional consequence of the mutant *WFS1* gene in vitro.

**Methods:**

A 27 years-old Chinese man was diagnosed as wolfram syndrome type 1 based on clinical data and laboratory data. DNA sequencing of *WFS1* gene and mitochondrial m.3337G > A, m.3243A > G mutations were performed in the patient and his 4 family members. Functional analysis was performed to assessed the in vitro effect of the newly identified mutant. ER stress were evaluated by ER stress response element (ERSE)-luciferase assay. Cell apoptosis were performed by CCK-8, TUNEL staining and flow cytometric analysis.

**Results:**

A novel heterozygous 10-base deletion (c. 2067_2076 del10, p.W690fsX706) was identified in the patient. In vitro studies showed that mutant p.W690fsX706 increased ERSE reporter activity in the presence or absence of thapsigargin instead of wild type *WFS1*. Knockdown of *WFS1* activated the unfolded protein response (UPR) pathway and increased the cell apoptosis, which could not be restored by transfection with *WFS1* mutant (p.W690fsX706) comparable to the wild type *WFS1*.

**Conclusions:**

A novel heterozygous mutation of *WFS1* detected in the patient resulted in loss-of-function of wolframin, thereby inducing dysregulated ER stress signaling and cell apoptosis. These findings increase the spectrum of *WFS1* gene mutations and broaden our insights into the roles of mutant *WFS1* in the pathogenesis of WS.

**Supplementary Information:**

The online version contains supplementary material available at 10.1186/s12902-021-00748-z.

## Introduction

Wolfram syndrome (WS, MIM 222300), also known as DIDMOAD syndrome, is an inherited autosomal recessive disease associated with diabetes insipidus (DI), insulin-dependent diabetes mellitus (DM), optic atrophy (OA) and deafness (D) [1]. Additional characteristics include neurological and psychiatric abnormalities, urodynamic disorders and endocrinological impairment [2, 3]. WS is a rare, monogenic disease with the prevalence of 1:770000 and a carrier frequency of 1:354 [2]. However, the prognosis is poor. Death occurs at the median age of 39 years with a major cause represented by respiratory failure as a consequence of neurological disabilities [4].

WS-1, the most common form of WS, is caused by mutations in the *WFS1* gene. The WS-1-associated gene, *WFS1*(OMIM 606201), maps to chromosome 4p16.1 and consists of eight exons (coding sequences is from exon 2 to exon 8), encoding an 890 amino-acid glycoprotein (wolframin) [5]. It has been shown that *WFS1* has played an important role in the negative regulation of ER stress signaling network and prevents cells (e.g., pancreatic beta cells) from apoptosis [6]. Consequently, therapeutics targeting the *WFS1* gene is pivotal towards potential treatments for WS spectrum disorders. Currently, over 200 distinct mutations of *WFS1* have been identified which considered to be the major mechanism underlying the development of the symptoms in WS [7]. Previous case report found some pathogenic variants of *WFS1*(p.Trp314Arg, p.Leu829Pro), particularly dominant variants, cause deafness or diabetes alone [8, 9]. Other dominant *WFS1* variants (p.Aal684Val, p.Glu462Gly) give rise to deafness and optic nerve atrophy or autosomal dominant congenital cataracts [10, 11]. Moreover, a novel nonsynonymous variant in *WFS1* associated with autosomal dominant DM without other features of WS [9]. These findings suggest that there may be a spectrum of phenotypes associated with WS based on differences in functional implications of the mutations. Therefore, further functional studies looking at the evolution of disease in patients with this rare syndrome will be needed to precisely define a genotype–phenotype relationship, thus contributing to early diagnosis and comprehensive genetic counseling to achieve future effective therapy.

In this study, we performed a clinical and genetic investigation on a Chinese male patient with clinically defined WS-1 and his families. Genetic analysis revealed a novel heterozygous 10-base deletion (c.2067_2076 del10, p.W690fsX706) in exon 8 of the *WSF1* gene. Further in vitro analysis showed this mutation led to increase ER stress and cell apoptosis, strongly indicating that the mutation is disease-causing.

## Methods

### Clinical data

The present study included one proband and his 4 family members. The clinical data were compiled from available medical records from the First Affiliated Hospital of Sun Yat-Sen University. The diagnosis of WS was based on manifestation of DM, optic atrophy and other abnormalities associated with WS. Diagnostic criteria for diabetes mellitus was defined as having any one of the following: 1) fasting plasma glucose level ≥ 126 mg/dl, or 2) plasma glucose ≥200 mg/dl two hours after a 75 g oral glucose load as in a glucose tolerance test, or 3) symptoms of high blood glucose and casual plasma glucose ≥200 mg/dl [12]. Audiological evaluation included routine physical examination and audiography to determine hearing status. The ophthalmological test consisted of examination of fundus with slit lamp biomicroscopy, indirect funduscopy, visual acuity and brain Magnetic Resonance Imaging (MRI). DI was diagnosed through the water restriction test followed by arginine vasopressin administration [13]. Partial DI was defined when the maximal urine concentration was 300–800 mOsm/kg [13]. Urinary tract was investigated by using Magnetic resonance urography (MRU) and urodynamic study. Reduced flow rate and abnormal flow rate patterns were also taken into consideration in the diagnosis of detrusor external sphincter dyssynergia (DESD) [14]. This study was approved by ethics committee of the First Affiliated Hospital of Sun Yat-sen University and conducted according to the provisions of the Declaration of Helsinki. Written informed consent was obtained from the patient and his family members.

### Genomic DNA analysis

Blood samples were collected from the patient with Wolfram syndrome and his 4 healthy family members. Genomic DNA was extracted with QIAamp DNA Blood Midi kit (Qiagen, USA). For the molecular analysis of *WFS1* (RefSeq NM_006005.2), primers were designed and Polymerase chain reaction (PCR) was carried out to amplify the entire coding sequences, the first exon and 300 bp intron sequences located at the boundaries between introns and exons. Primer sequences and PCR conditions are available upon request. PCR product confirmation by electrophoresis and purification with QIAquick PCR Purification Kit (Qiagen, USA) were followed. The next step was Sanger sequencing with or without cloning, depends on the result of direct sequencing. The cloning was done with EZ-T™ Fast Ligation Kit (GenStar, China) and Trans1-T1 Phage Resistant Chemically Competent Cell (TransGen Biotech, China). The sequencing results were blast in NCBI against the wild type *WFS1* gene (NG_011700.1). The identified mutation was checked against databases of published polymorphisms and mutations (http://www.khri.med.umich.edu/research/lesperance_lab/low_freq.php;http://www.hgmd.cf.ac.uk/ac/index.php).

The Multiplex Ligation Dependent Probe Amplifification (MLAP) reactions were performed to screen all the exons of *WFS1* gene in the father using the SALSA MLPA kit P163 probe mix (MRC-Holland BV, Amsterdam, the Netherlands). DNA specimens from 2 healthy individuals (male and female) were used as normal controls, and for all reactions, the manufacturer-provided protocol was followed. MLPA was performed according to the manufacturer provided protocol. The PCR products obtained as a result of MLPA reaction were run on the Applied Biosystems 3500 capillary electrophoresis device. As a result of the process, peak images and peak areas of the probes of each sample were obtained in the CEQ program. Excel based Coffalyser (Coffalyser, MRC-Holland BV, Amsterdam, the Netherlands) program was used for analysis.

### Gene expression analysis

Total RNA was isolated from patient whole blood using the ipureTRizol blood RNA kit (IGE, Guangzhou, China). First-strand cDNA was synthesized from total RNA with an oligodT primer (First strand cDNA synthesis Kit,IGE, Guangzhou, China). Primers were designed and PCR was carried out to amplify the whole exon 8 (F 5′-CTTCTCTGTGGTGGGGATGG-3’and R 5′-TGCCCACGGTAATCTCAAAC-3′). The PCR products were gel purified (QIAquick gel extraction kit; QIAGEN) and directly sequenced on an ABI 3730 Automated DNA Sequencer (PE Applied Biosystems).

### Mitochondrial DNA mutation analysis

Mitochondrial mutation of m. 3337G > A is screened with the same method as the above mentioned. For the differential diagnosis with maternally inherited diabetes and deafness (MIDD), the mutation of mitochondrial tRNALeu (UUR) (m. 3243A > G) was also screened.

### Cell culture

HEK-293 T cells and HK-2 cells were obtained from the American Type Culture Collection (ATCC). HEK-293 T cells were cultured in Dulbecco’s Modified Eagle’s Medium (DMEM) (Gibco, Canada) containing 10% fetal bovine serum (FBS) (Life Technologies Corporation, USA). HK-2 cells at passages 5–10 were grown in complete DMEM-F12 media (Gibco) supplemented with 10% FBS. All media were supplemented with 100 U/mL penicillin and 100 μg/mL streptomycin (Gibco). Both cell types were cultured in an incubator at 37 °C under a humidified 5% CO_2_ atmosphere for experiments.

### Plasmids construction and ERSE-luciferase assay

Wild type (WT), p. W690fsX706 and p.F883X WFS1 cDNA were respectively synthesized and cloned into the pcDNA3.1 (+) vector by Generay Biotech (Shanghai, China). Sanger sequencing (ACGT, Wheeling, IL) was used to validate the sequence of the expression plasmids. HEK-293 T cells were seeded in 12-well plates, then transfected with 0.5 μg of ERSE-luciferase plasmid together with 1.0 μg of each WT, p.W690fsX706 or p.F883X WFS1 expression plasmid and 10 ng of pRL-TK vector (Promega, USA). After 24-h transfection, the cells were treated with or without thapsigargin (10 nmol/L) for 6 h. The assay was performed using the dual-luciferase reporter assay system (Promega, USA) according to the manufacturer’s instructions, and luciferase activities were normalized to Renilla luciferase values of the co-transfected pRL-TK vector.

### Stable cell lines and cell transient transfection

PLKO.1-based shRNA plasmid targeting *WFS1* (shWFS1) and scrambled shRNA plasmid (shControl) were purchased from Generay Biotech (Shanghai, China). The targeted sequence was: 5′**-**GACGA CGAAGATGATGACGAGCTGG-3′. Lentivirus infection was carried out following the manufacturer’s protocol. In brief, constructs were amplified in HEK-293 T cells, and the viral supernatants were collected, added to the HK-2 cells and incubated for 24 h. Then, puromycin was added to the HK-2 medium to select for the cells with stable viral integration. HK-2 cells with stable knockdown of *WFS1* were respectively transfected with wild type (WT), p.W690fsX706, p.F883X *WFS1* expression plasmids and empty vector using Lipofectamine 3000 transfection reagent (Invitrogen) according to the manufacturer’s instructions. After 24 h or 48 h of transfection, the cells were harvested for qRT-PCR and western blotting analysis.

### RNA extraction and qRT-PCR assay

HK-2 cells were harvested using TRIzol reagent (Invitrogen) and total RNA was extracted using ethanol precipitation. 1 μg of total RNA from cells was then reverse transcribed using PrimeScript RT Reagent Kit with gDNA Eraser (TaKaRa, Japan). qRT-PCR reactions were performed on ABI 7500 real-time PCR system (Applied Biosystems, USA) with TB Green Premix Ex Taq II (TaKaRa, Japan). The expression levels of genes were determined by 2^-ΔΔCt^ methodology, normalized against β-actin. The primer sequences used for qRT-PCR were as follows: human β-actin, 5′-CATGTACGTTGCTATCCAGGC-3′ (F) and 5′-CTCCTTAATGTCACGCACGAT-3′ (R); human WFS1, 5′-AGAACGAACGGGAGGTGA-3′ (F) and 5′-TCTTGGACTCGCTGCTGA-3′ (R); human GRP78, 5′-CATCACGCCGTCCTATGTCG-3′ (F) and 5′-CGTCAAAGACCGTGTTCTCG-3′ (R); human XBP1, 5′-GGATTCTGGCGGTATTGA-3′ (F) and 5′-AAAGGGAGGCTGGTAAGG-3′ (R); human CHOP, 5′-AGAACCAGGAAACGGAA ACAGA-3′ (F) and 5′-TCTCCTTCATGCGCTGCTTT-3′ (R).

### Western blotting

HK-2 cells were lysed in ice-cold RIPA lysis and extraction buffer (Thermo Scientific, USA) containing a protease inhibitor cocktail (Promega, USA). Protein samples (30 μg protein equivalent) were electrophoresed using 10% SDS-PAGE and transferred to polyvinylidene fluoride (PVDF) membranes (Invitrogen). The membranes were blocked with 5% milk for 1 h on an orbital shaker, and then incubated overnight at 4 °C with primary antibodies. The antibodies used in the study were as follows: anti-β-Actin (rabbit, 1:1000; Cell Signaling), anti-WFS1 (rabbit, 1:1000; Cell Signaling), anti-BiP (rabbit, 1:1000; Cell Signaling), anti-ATF6 (rabbit, 1:1000; Cell Signaling), anti-XBP1 (rabbit, 1:1000; Cell Signaling) and anti-CHOP (mouse, 1:1000; Cell Signaling). The membranes were washed for three times, followed by incubation with horseradish peroxidase-conjugated secondary antibody (goat anti-rabbit, 1:10000, [Abcam] or goat anti-mouse, 1:10000, [Abcam]). Enhanced chemiluminescence reagents (Millipore, USA) were used to detect antigen–antibody complexes and the Bio-Rad electrophoresis image analyzer (Bio-Rad, USA) were used to visualize specific bands.

### Cell viability assay

Stable HK-2 cells (shControl or shWFS1) were seeded in 96-well plates at 1 × 10^4^ cells per well (five replicate wells) and transfected with indicated expression plasmids. After 48 h of transfection, cell viability was determined using Cell Counting Kit-8 (CCK-8) (Dojindo, Japan) following the manufacturer’s protocol. OD values were obtained using microplate spectrophotometer at 450 nm wavelength.

### TUNEL staining assay

Stable HK-2 cells (shControl or shWFS1) were seeded in 6-well plates at 5 × 10^5^ cells per well, and transfected with expression plasmids respectively. TUNEL staining was performed at 48 h post-transfection using the one step TUNEL apoptosis assay kit (Beyotime Biotech, China) according to the manufacturer’s instructions. Images were captured by microscope and results were analyzed by ImageJ (ImageJ 1.46r, USA).

### Flow cytometric analysis of cell apoptosis

The HK-2 cells were harvested after 48-h transfection and double-stained with Annexin V and propidium iodide (PI) dye by using Annexin V-FITC/PI apoptosis assay kit (Lianke Biotech, China) following the manufacturer’s protocol. The fluorescent intensity was measured by a FACSCalibur™ flow cytometry (BD Biosciences, USA) and data were analyzed by Flow Jo v.10.0.7 software.

### Statistical analysis

The results were presented as mean ± SEM from at least three independent experiments. Student’s *t* test was used for the comparison between two groups and One-way ANOVA (followed by Tukey’s post hoc test) was used for comparing more than two groups. All statistical analyses were conducted using SPSS v.22.0 (SPSS Inc., USA). A *P* value < 0.05 was considered statistically significant.

## Result

### Clinical manifestation of the patient with WS

The patient, a 27 years-old Chinese male, was diagnosed wolfram syndrome based on the minimal criteria of juvenile onset diabetes and optic atrophy and also with other abnormalities associated with WS-1. The patient was diagnosed type 1 diabetes mellitus with ketoacidosis at age 6 and blood glucose was controlling with insulin four times each day (Insulin Glargine Injection once daily and Insulin Aspart Injection three times a day before each meal). No regular blood glucose was checked up after the diagnosis, but several episodes of hypoglycemia did occur. Some related examinations are done in our hospital and is summarized in Table 1. An unsatisfied median glaciated hemoglobin (HbA1C) and a low concentration of C-peptide even after arginine infusion reveal poor function of beta cells with poor control of blood glucose. Despite modifications in diet, exercise, and medications in our center, the patient still had wide fluctuations in his blood glucose levels. Therefore, we started him on an insulin pump and continuous glucose monitoring showed flatter curve of the blood glucose and no hypoglycemia attack afterward.

**Table 1:** Patient’s clinical manifestation

Phenotype	Emerge age	Relevant examination results
Diabetes Mellitus (insulin dependent)	6 years old	The level of fasting blood glucose was fluctuated from 8 to 10 mmol/L while the arrival at our center; HbA1c: 8.00%; Release of C-peptide after arginine infusion: 0 min➔0.013 nmol/L (0.039 ng/ml); 2 min➔0.035 nmol/L (0.105 ng/ml); 3 min➔0.037 nmol/L (0.111 ng/ml); 5 min➔0.036 nmol/L (0.110 ng/ml); Glutamic acid decarboxylase (GAD) antibodies (+).
Sensorineural deafness	14 years old	Pure tone average hearing level: 60-65 dB; Air bone gap≦10 dB; Acoustic impedance audiometry: type A tympanogram of both sides; Contralateral and ipsilateral acoustic stapedius reflex positive on both sides.
Optic nerve atrophy	19 years old	Visual acuity: count fingers at 15 cm (bilateral); Funduscopy: pallor retina and optic nerve with unclear cup to disc ratio; Brain MRI: bilateral optic nerve atrophy.
neurogenic bladder	21 years old	MRU showed bilateral dilated urinary tract; Urodynamic study: detrusor external sphincter dyssynergia (DESD).
Partial central diabetes insipidus	21 years old	Urinary osmolality: 95.0 mOsm/kg; Plasma osmolality: 291 mOsm/L; Urinary gravity: 1.002; Water-deprivation and vasopressin test showed partial central diabetes inspidus.

**Fig. 1:**
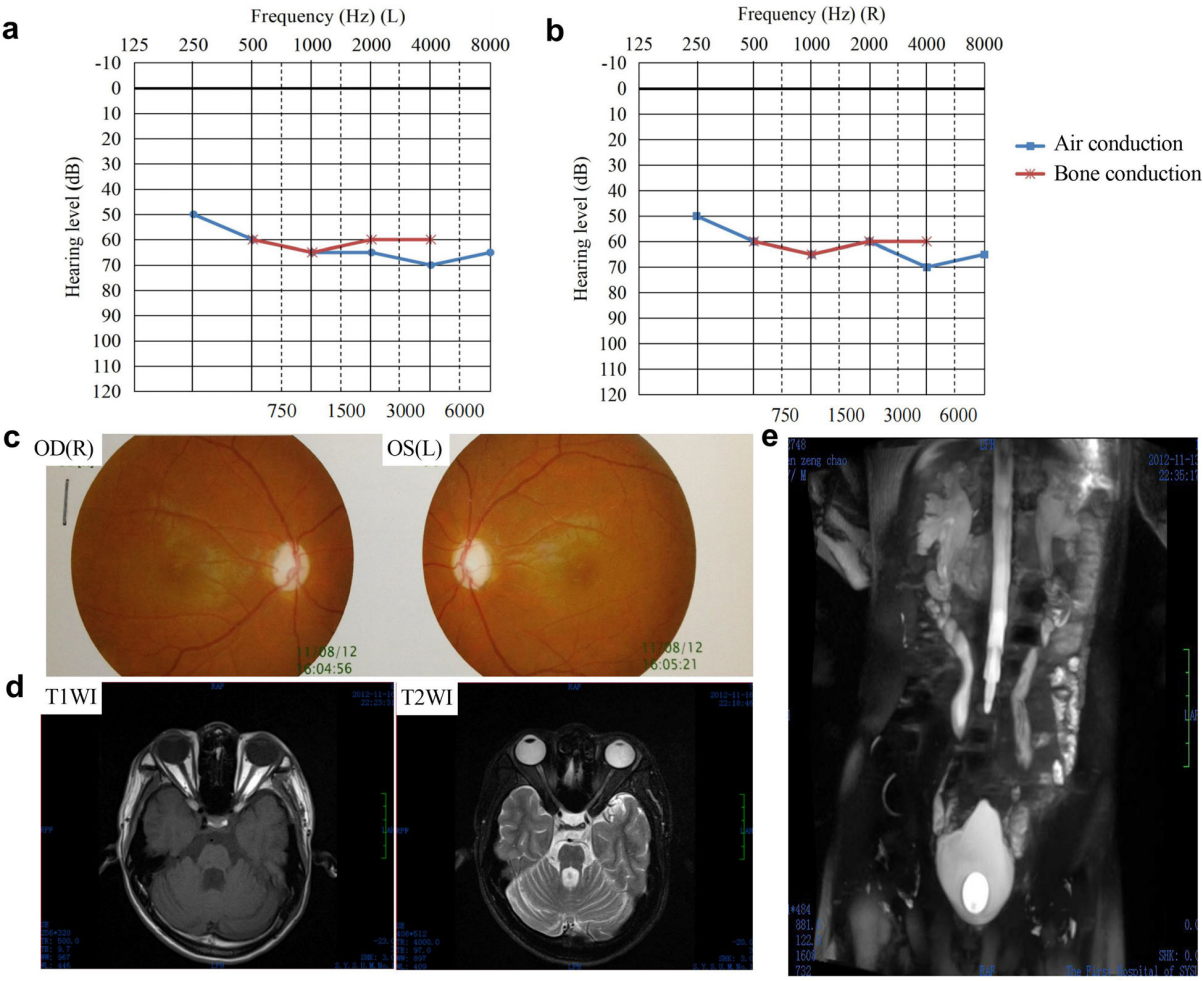
The patient presented with hearing loss, decreased vision, optic atrophy and neurogenic bladder. **a** Audiograms show left sensorineural hearing loss. **b** Audiograms show right sensorineural hearing loss. **c** Fundus image shows atrophic optic discs with temporal pallor. **d** Axial brain MRI shows bilateral optic nerve atrophy on T1WI (left) and T2WI (right). **e** MRU shows marked dilation of bilateral renal pelvis, calyces and ureter

The patient complained progressive hearing loss since age of 14 and our test showed severe loss with a pure tone average hearing level of 60-65 dB. Both the high frequency and low frequency were affected but the former was slightly profounder. Air bone gap≦10 dB also suggested sensorineural deafness (Fig. 1a, b). Progressive decreased vision started from age 19. On examination, his visual acuity was count fingers at 15 cm in both eyes. The pupils were mydriatic with decreased light reflex. Funduscopy exhibited pallor retina and optic nerve with unclear cup to disc ratio (Fig. 1c). Brain MRI showed narrow optic nerve of both sides (Fig. 1d). The patient had frequcent, urgent and difficulty of urination and was diagnosed as neurogenic bladder with urinary tract infection at age 21. Foley catherter was used to keep the bladder empty. MRU showed bilateral dilated pelvis, calyx and ureter (Fig. 1e); and urodynamic study suggested detrusor external sphincter dyssynergia (DESD). Recurred polydipsia and diuresis at age 21. The investiagtion results of low urinary osmolality (95.0 mOsm/kg), relative high plasma osmolality (291 mOsm/L) and low urinary gravity (1.002), as well as the significant increased urinary osmolality after water deprivation and well respond to the followed arginine vasopressin revealed partial central diabetes inspidus. The patient’s siblings as well as his non-consanguineous parents had regular primary health care, no similar symptoms or abnormal blood glucose level could be found.

### Mutation analysis of *WFS1*

**Fig. 2:**
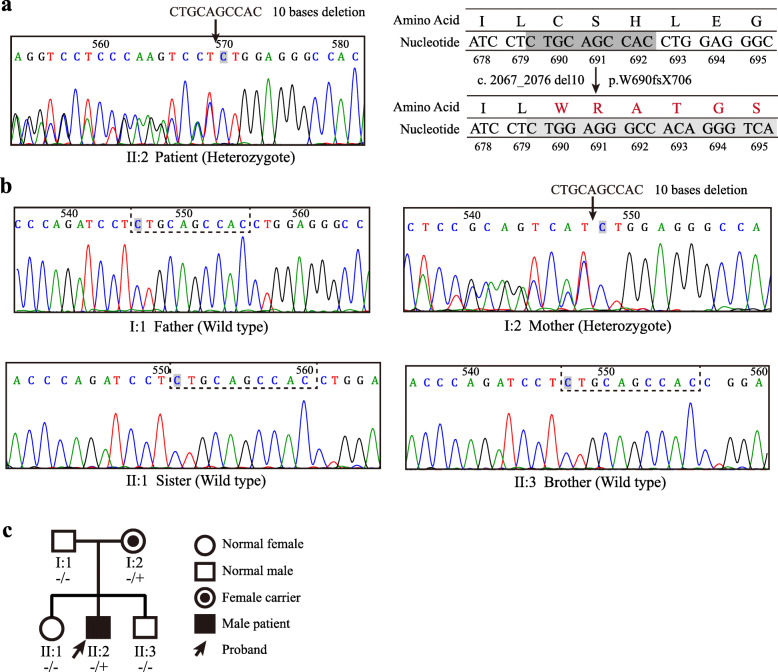
PCR-direct sequencing of *WFS1* and the pedigree of the patient’s family. **a** Sequence chromatograms of *WFS1* exon 8 in the patient and his families. Reverse sequencing of *WFS1* exon 8 in the patient indicated the presence of heterozygous 10-bp deletion mutation. Arrow indicates the deletion site. Double bands appear after the deletion site. This deletion resulted in a frame shift mutation starting at codon 690, and a terminator (UGA) was generated at position 706. **b** The heterozygous 10-bp deletion in exon 8 was also found in the patient’s mother. Forward sequencing of *WFS1* exon 8 revealed that his father, sister and brother did not have this mutation. **c** The pedigree of the patient’s family. The first line below each symbol represents generation and identification number. -, absence of *WFS1* mutation; +, presence of *WFS1* mutation

To further confirm the diagnosis, we performed sequence analysis of the patient *WFS1*. DNA-sequencing patterns were compared to the *WFS1* cDNA sequence in the GenBank (NCBI Reference Sequence: NG_011700.1; Gene ID: 7466). A summary of the found variants in *WFS1* was shown in Table 2. A de novel heterozygous 10-base deletion in exon 8 (c. 2067_2076 del10) was identified. This deletion resulted in a frame-shift mutation starting at codon 690, and a terminator (UGA) was generated at position 706 (p.W690fsX706), thus reducing the number of amino acids from the 690 to 706 (Fig. 2a). Details of pathogenicity analysis were provided in Supplementary Fig. S1. This mutation was absent from the Human Gene Mutation Database (HGMD) and the database of mutations and polymorphisms in the *WFS1* gene of the Kresge Hearing Research Institute (KHRI). We then analyzed the genomic DNA of the parents, as well as his sister and brother. Surprisingly, the same heterozygous mutation in exon 8 was also found in the patient’s mother, who had no signs or symptoms at that time. His father, sister and brother did not have this mutation (Fig. 2b). However, the pedigree of the patient’s family suggested that the inheritance of Wolfram syndrome in this patient did not conform to autosomal recessive inheritance (Fig. 2c). Furthermore, we performed the cDNA sequencing which identified the same heterozygous ten bases deletion in exon 8 (c. 2067_2076 del10) as the result of the genomic DNA sequencing in patient and his mother (Supplementary Fig. S2). Next, to rule out the possible large deletions in the father, we analyzed *WFS1* gene by using Multiplex ligation dependent probe amplifification (MLPA) method. The results showed no large deletions/duplication in all the exons of *WFS1* gene in the father (see Supplementary Fig. S3). To clarify the reasons why the same mutation in the coding region of *WFS1* gene of patient and his mother induced obviously different phenotypes, we also analyzed the non-coding regions of *WFS1* gene including flanking sequences and junctions between exons and introns. The results showed that no pathogenic mutations were found in the non-coding region of *WFS1* gene of the patient and his families (Table 3). In addition, we screened mitochondrial mutations of m.3337G > A and m.3243A > G, which were associated with WS and maternally inherited diabetes and deafness (MIDD), respectively [15, 16]. Both mutations were not found in the patient and his families (Supplementary Fig. S4).

**Table 2:** A summary of the found variants in all exons of *WFS1* gene in this study

Region	Nucleotide changes	Amino acid changes	Types of variation	Status	Subjects
Exon 5	*c. 684 C > G*	*No change*	Silent mutation		All members
Exon 8	*c. 997 G > A*	*V333I*	Missense mutation	polymorphism	All members
Exon 8	*c. 1185 C > T*	*No change*	Silent mutation		All members
Exon 8	*c. 1500 C > T*	*No change*	Silent mutation		All members
Exon 8	*c. 1832 G > A*	*R611H*	Missense mutation	polymorphism	All members
**Exon 8**	***c. 2067_2076 del 10***	***W690fsX706***	**Frameshift mutation**	**Heterozygous (novel)**	**I-2(mother), II-2 (patient)**
Exon 8	*c. 2433 G > A*	*No change*	Silent mutation		All members
Exon 8	*c. 2565 A > G*	*No change*	Silent mutation		All members

**Table 3:** A summary of the found variants in the non-coding regions of *WFS1* gene including flanking sequences and junctions between exons and introns in this study

Region	Nucleotide changes	subjects
5′-Flanking sequence	*g.-258_-253del6*	All members
Intron 3	*c.486–168 T > C*	All members
Intron 4	*c.630 + 147 C > G*	All members
	*c.631–279 G > C*	All members
	*c.631-161_159del14*	All members
Intron 5	*c.801 + 144 C > G*	All members
	*c.801 + 257 T > C*	All members
	*c.802–196 T > C*	All members
	*c.802–169 A > G*	All members
Intron 6	*c.882 + 63 T > C*	I-2(mother)
	*c.882 + 114 A > G*	All members
	*c.882 + 131 C > T*	All members
	*c.882 + 195 C > G*	All members
	*c.882 + 277 G > A*	All members
	*c.882 + 367 G > A*	I-2(mother), II-2 (patient)
	*c.882 + 371 A > G*	All members
	*c.883-321G > T* ^a^	I-1(father), II-2 (patient) and II-3 (brother)
Intron 7	*c.1032–164 C > G*	All members
	*c.1032–185 C > T*	All members

^a^polymorphism, Reference: Kawamoto et al. (2004)

### The novel *WFS1* mutation (p.W690fsX706) increases ER stress

**Fig. 3:**
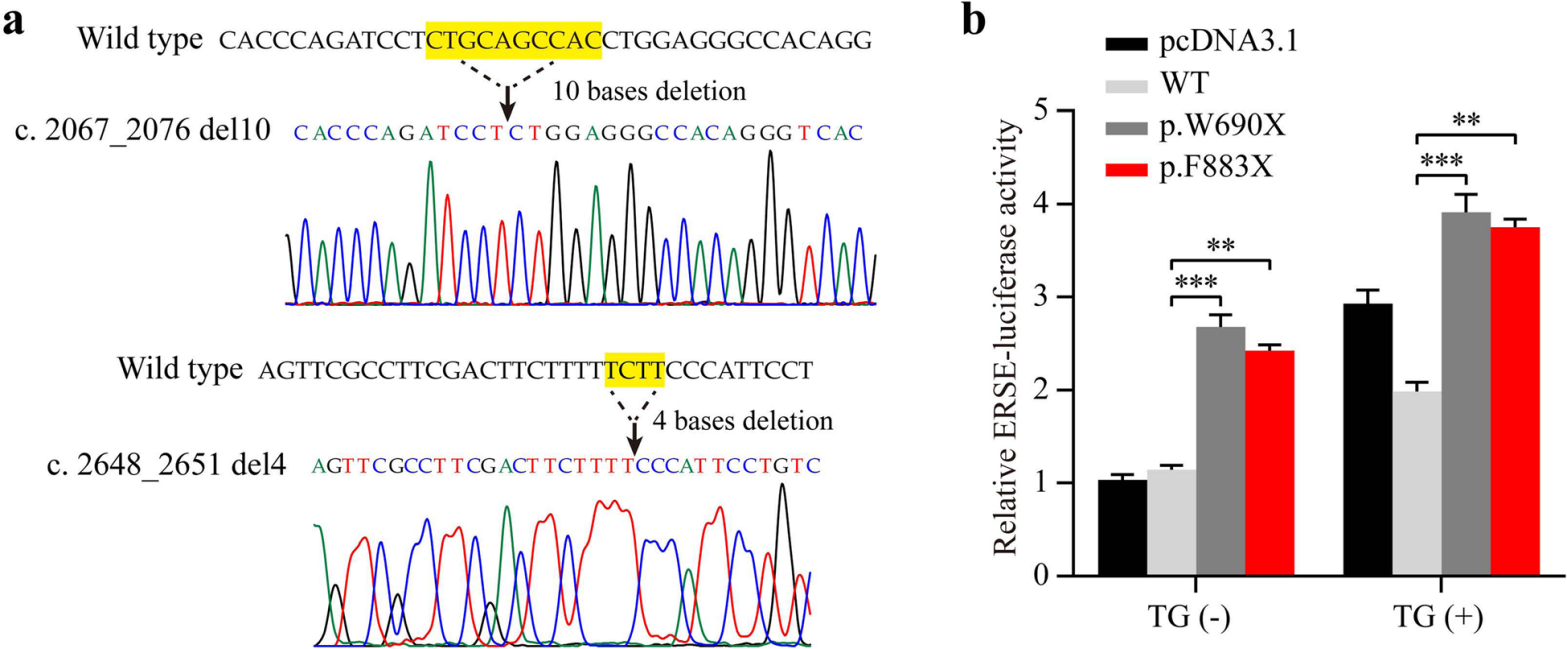
Luciferase reporter assays in HEK293T cells. **a** Sanger sequencing was used to verify the sequence of mutant (c. 2067_2076 del10, p.W690fsX706) *WFS1* and mutant (c. 2648_2651 del4, p.F883fsX950) *WFS1* expression plasmids. **b** HEK293T cells were transfected with the ERSE reporter vector together with the control (pcDNA3.1), wild type *WFS1* (WT), mutant p.W690fsX706 (p.W690X) *WFS1*, or the positive control mutant p.F883fsX950 (p.F883X) *WFS1* expression plasmid. Cells were treated with or without thapsigargin (TG, 10 nmol/L) for 6 h and the relative luciferase intensity was measured. The results were normalized to *Renilla reniformis* luciferase activity. Data were presented as mean ± SEM. One-way ANOVA, Tukey’s post-hoc test, **P < 0.01 and ***P < 0.001

*WFS1* gene encodes a 100-kDa protein called wolframin, which is predominantly localized in the endoplasmic reticulum and involved in ER stress [17, 18]. To determine whether the novel *WFS1* mutation (p.W690fsX706) could cause the dysregulation of the ER stress response, we examined the effect of the p.W690fsX706 mutation and other *WFS1* mutation c. 2648_2651 del4 (p.F883fsX950), which was identified as a novo mutation in two unrelated individuals with WS [19, 20]. Mutant (p.W690fsX706) *WFS1* and mutant (p.F883fsX950) *WFS1* expression plasmids were constructed, and Sanger sequencing was used to verify the sequence of these plasmids (Fig. 3a). Then, HEK293T cells were transfected with the ERSE reporter vector together with control (pcDNA3.1), wild type *WFS1* (WT), mutant p.W690fsX706 (p.W690X) *WFS1*, and mutant p.F883fsX950 (p.F883X) *WFS1* expression plasmids, respectively. Regardless of the presence or absence of the ER stress inducer thapsigargin, both mutants p.W690fsX706 and p.F883fsX950 induced stronger ERSE reporter activity than wild type *WFS1*, which suggested that the patient mutant p.W690fsX706 may be pathogenic by increasing ER stress (Fig. 3b).

### *WFS1* mutation (p.W690fsX706) leads to loss-of-function of wolframin

**Fig. 4:**
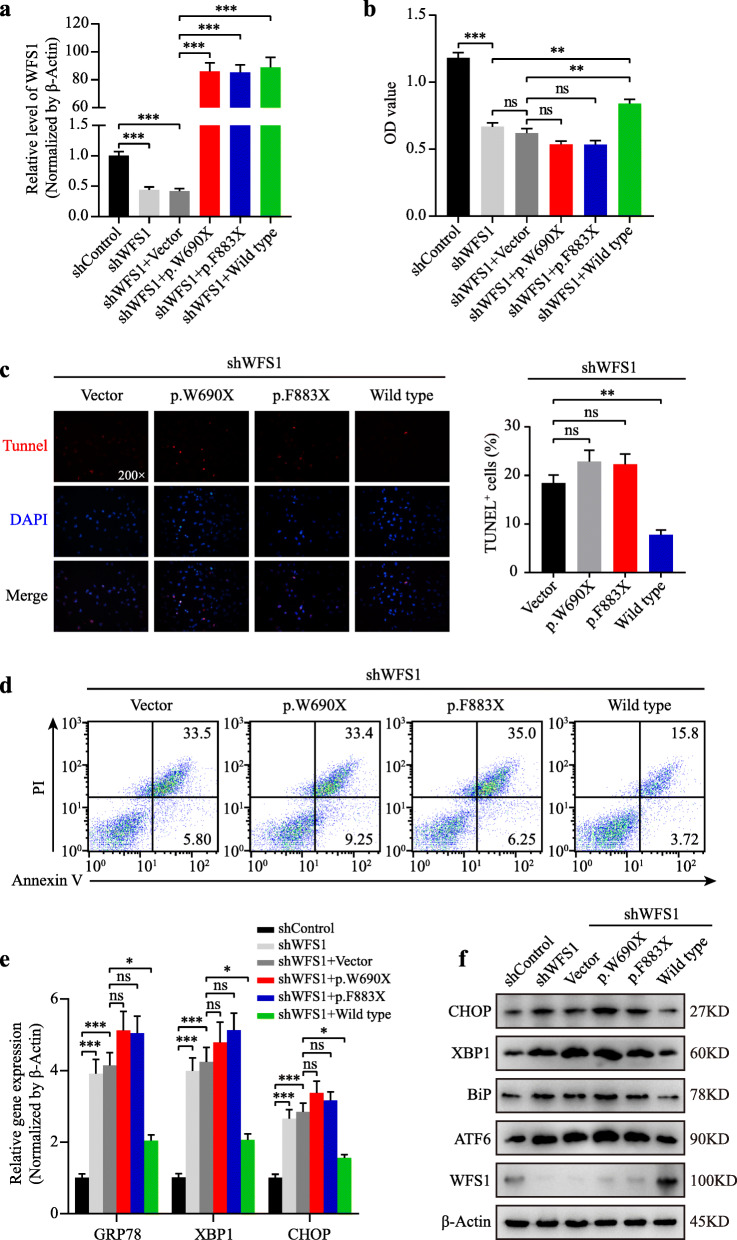
*WFS1* mutation (p.W690fsX706) induces cell apoptosis by increasing ER stress. HK-2 cells with stable knockdown of *WFS1* were respectively transfected with wild type (WT), p.W690fsX706 (p.W690X), p.F883fsX950 (p.F883X) *WFS1* expression plasmids and empty vector. **a** Relative expression level of *WFS1* was verified by qRT-PCR in HK-2 cells after indicated interventions. **b** At 48 h post-transfection, cell viability of HK-2 cells was measured by CCK-8 assay. **c** After 48 h of transfection, TUNEL staining of HK-2 cells was carried out, and representative images were shown individually and merged. TUNEL-positive HK-2 cells were calculated. Magnification: 200X. **d** After transfection with indicated expression plasmids for 48 h, HK-2 cells stained with Annexin V and propidium iodide (PI) were analyzed by flow cytometry for cell apoptosis assessment. **e** Relative mRNA levels of ER stress-related markers (GRP78, XBP1 and CHOP) were measured by qRT-PCR. **f** The levels of *WFS1* protein and ER stress-related proteins (ATF-6, BiP, XBP1 and CHOP) in HK-2 cells were analyzed by western blotting. β-Actin served as a loading control. Values were represented as mean ± SEM. One-way ANOVA, Tukey’s post-hoc test, *P < 0.05, **P < 0.01 and ***P < 0.001. ns means no statistical significance

Wolframin protein is involved in the suppression of ER stress-induced cell apoptosis by negatively regulating ATF6α and the unfolded protein response (UPR) pathway [17], raising the possibility that the p.W690fsX706 mutant may lose its negative regulation of the ER stress pathway, thereby inducing cell apoptosis and Wolfram syndrome. To test this hypothesis, we constructed stable *WFS1*-knockdown HK-2 cells and then transfected cells with wild type (WT), p.W690fsX706, p.F883fsX950 *WFS1* expression plasmids and empty vector, respectively. qRT-PCR analysis was performed and the fragment amplified by the *WFS1* primer was the same in cells transfected with both mutant and WT *WFS1* expression plasmids. Compared with the control group, *WFS1* mRNA expression levels were markedly decreased in shWFS1 group, and the mRNA levels of *WFS1* were significantly increased after transfecting wild type, p.W690fsX706 and p.F883fsX950 *WFS1* expression plasmids (Fig. 4a). CCK8 assay showed that *WFS1* silencing-mediated decrease in cell viability was significantly restored by transfection with WT *WFS1* expression plasmid, whereas both mutant *WFS1* expression plasmids failed to improve cell viability (Fig. 4b). Although the difference was not statistically significant, it seemed that the viability of the cells transfected with mutant *WFS1* expression plasmids was lower than that of the empty vector. Similarly, TUNEL assay and flow cytometry analysis illustrated that transfection with WT *WFS1* expression plasmid obviously decreased cell apoptosis, compared with two mutant *WFS1* expression plasmids (Fig. 4c and d). Moreover, knocking down *WFS1* significantly up-regulated the mRNA levels of ER stress-related molecules (GRP78, XBP1 and CHOP), which could be restored by transfection with WT expression plasmid instead of two mutant *WFS1* expression plasmids (Fig. 4e). Consistently, *WFS1* silencing-mediated increase in ER stress-related protein levels (ATF6, BiP, XBP1 and CHOP) were reduced by transfection with WT expression plasmid instead of two mutant *WFS1* expression plasmids (Fig. 4f). Besides, the *WFS1* protein levels in shWFS1 group were obviously knocked down. Despite the increased mRNA levels of *WFS1* in cells respectively transfected with two mutant and WT *WFS1* expression plasmids, transfection with WT *WFS1* expression plasmid could restore the protein levels of *WFS1*, while two mutant *WFS1* expression plasmids could not (Fig. 4a and f). These results indicated that the *WFS1* mutation (p.W690fsX706) cause loss-of-function, leading to dysregulated ER-stress and cell apoptosis.

## Discussion

In the current study, we identified a novel heterozygous mutation of *WFS1* (c. 2067_2076 del10, p.W690fsX706) in a patient with WS. Moreover, in vitro analysis suggested that this *WFS1* mutation resulted in loss-of-function of wolframin protein, thus increasing ER stress and cell apoptosis, supporting the conclusion that the mutation is disease-causing.

Wolfram syndrome (WS) is a rare autosomal recessive neurodegenerative disorder with an early age of onset, a high morbidity rate, and a poor prognosis [21]. Because WS affects multiple organ systems and has variable presentation, it is easy to miss and misdiagnose. The development of WS is chronic and progressive, and the most frequent clinical manifestation is early onset diabetes, which should be distinguished from type 1 diabetes [22]. WS is generally believed to have no autoimmune factors involved and most autoantibodies are negative. On the contrary, most type 1 diabetes patients have positive autoantibodies, but there are few cases of Wolfram syndrome combined with positive autoantibodies, such as our patient in this study (GAD antibody positive) and the case report of a patient in Japan (GAD antibody positive, IA-2 antibody positive) [23]. Moreover, compared with type 1 diabetes, WS have a low prevalence of ketoacidosis and rare microvascular complications, slower disease progression, and a higher incidence of severe hypoglycemia. Consequently, it is very important to detect and diagnose patients with WS as early as possible. With the progression of WS, the optic atrophy began to appear, mostly after the onset of diabetes [24]. Although the manifestation of optic atrophy is blurred vision, it needs to be distinguished from diabetic retinopathy. Furthermore, patients with WS has many clinical similarities with MIDD and MELAS (Mitochondrial Encephalomyopathy, Lactic Acidosis and Stroke-like episodes syndrome), making the diagnosis more difficult. Therefore, it is essential to perform genetic analysis to confirm the diagnosis of WS.

It is reported that *WFS1* is the main pathogenic gene and most cases of WS harbored heterozygous or compound heterozygous *WFS1* mutations [25]. *WFS1* gene encodes an 890 amino-acid glycoprotein (wolframin), which is a transmembrane protein localized to the ER [18]. The C-terminal of wolframin protein is conserved in rats, mice and humans, and Smurf1 regulated the degradation of wolframin by binding to the 667–700 amino acids at the C-terminal [25]. Besides, deletion of the last seven amino acids at the C-terminal of wolframin could provoke all clinical symptoms of WS [20]. All these studies implied the importance of the C-terminal. In this study, we identified a novel *WFS1* mutation resulting in a frame-shift mutation starting at codon 690 and a premature stop codon at position 706 (p.W690fsX706), which led to mutation and truncation of the C-terminal of wolframin (186 amino acids reduced). Accordingly, it is reasonable to assume that this novel mutation can affect the structure and function of wolframin. Accumulating evidence indicated that *WFS1* was a component of the unfolded protein response (UPR) that could mitigate ER stress response, and high levels of ER stress signaling in affected cells contributed to β-cell death and neuronal cell dysfunction in WS [26, 27]. *WFS1* negatively regulated ATF6α and the activation of the ER stress response element (ERSE) could be attenuated by *WFS1* expression [28]. *WFS1* mutants activated ER stress target genes such as CHOP, GRP78 /Bip and XBP-1, leading to cell dysfunction [29]. Our in vitro studies demonstrated that knockdown of WFS1 was accompanied by activation of components of the UPR. Moreover, compared with WT, *WFS1* mutant (p.W690fsX706) lacked the ability to suppress the hyperactivation of the UPR and rescue the cell apoptosis induced by silencing *WFS1*. Based on these findings, loss-of-function of our *WFS1* mutant might be one of the causes of WS in this patient.

Although most *WFS1* mutations in WS patients were detected on both alleles and the inheritance of WS was considered to be autosomal recessive [18]. Increasing studies have reported a single heterozygous *WFS1* mutation in WS patients. For example, a heterozygous mutation of *WFS1* (p.N325_I328del), which could cause constitutive ER stress, was found in a patient with WS [28]. Autosomal dominant DM without other features of WS in a large family was associated with a heterozygous *WFS1* mutation (p.W314R) [9]. The patient in our study had typical clinical manifestations of WS and was heterozygous for p.W690fsX706 of *WFS1*. Interestingly, the patient’s mother had the same heterozygous mutation in exon 8 of *WFS1* gene without any signs or symptoms at that time. Notably, it has been reported that a single heterozygous *WFS1* mutation was found in patients with WS, and the same mutation could be found in family members without disease [30, 31]. Generally speaking, the reasons may include mutations in non-coding regions, differences in transcription or translation of *WFS1* gene, mutations in mitochondrial genes, and the influence of other genes or environmental factors. Giuliano et al. detected a mutation in the intron 7 of *WFS1* gene, resulting in changes in transcription and significantly truncated protein [19]. Elli et al. found mutations in intron 1 and the promoter of *WFS1* gene in a patient with WS, leading to a variant with the exon 2 deleted and reducing wolframin protein levels [32]. Since flanking sequences and the intron sequences on both sides of exons played more important roles in the functions of the non-coding regions of *WFS1* gene, we analyzed these regions in the patient and his families, and did not find pathogenic mutations. Furthermore, the mutant protein was expressed in substantial quantities in vitro, suggesting it is not subjected to non-sense mediated decay or degradation. The results suggest that in addition to mutations in gene sequences, differences in gene expression regulation (the transcription, mRNA processing and translation) as well as the epigenetic regulation including DNA methylation and genomic imprinting may also contribute to WS, which requires further investigation. Moreover, mitochondrial mutations m.3337G > A and m.3243A > G, which were respectively associated with WS and MIDD, were not found in the patient and his families. Of note, a single heterozygous *WFS1* mutation (p.Arg703Cys) was identified in a family with diabetes diagnosed early (14 years) and late (55 and 60 years) in life [33]. Furthermore, atypical dominant and recessive cases have been described recently [34, 35]. In our study, it is possible that the patient’s mother has a later age of onset or fewer clinical symptoms, which needs to be confirmed by follow-up. Although *WFS1* gene is thought to be the pathogenic gene of WS in our patient, we cannot completely rule out the influence of other genes and environmental factors, which may cooperate *WFS1* mutation to cause WS.

## Conclusions

Taken together, we reported a novel heterozygous mutation of *WFS1* resulting in loss-of-function of wolframin, thereby inducing dysregulated ER stress signaling and cell death. These findings increase the spectrum of *WFS1* gene mutations and broaden our insights into the roles of mutant *WFS1* in the pathogenesis of WS.

## Supplementary Information

**Additional file 1.**

**Additional file 2.**

**Additional file 3.**

**Additional file 4.**

## Data Availability

The data that support the findings of this patient and his family are available in the GenBank (accession numbers: MT832010-MT832014).

## Abbreviations

ATCC: American type culture collection
D: Deafness
DESD: Detrusor external sphincter dyssynergia
DI: Diabetes insipidus
DMEM: Dulbecco’s modified Eagle’s medium
DM: Diabetes mellitus
ER: Endoplasmic reticulum
ERSE: Endoplasmic reticulum stress response element
FBS: Fetal bovine serum
MIDD: Maternally inherited diabetes and deafness
MRU: Magnetic resonance urography
OA: Optic atrophy
PCR: Polymerase chain reaction
UPR: Unfolded protein response
WS: Wolfram syndrome
*WFS1*: Wolfram syndrome 1
