# Supplementary material for: A novel mutation of WFS1 gene leading to increase ER stress and cell apoptosis is associated an autosomal dominant form of Wolfram syndrome type 1
Source: BMC Endocr Disord. 2021 Apr 21;21:76. doi: 10.1186/s12902-021-00748-z (PMC8059287; doi:10.1186/s12902-021-00748-z)
Supplement: Supplementary file 1 — Additional file 1. [file 12902_2021_748_MOESM1_ESM.pdf]

A novel mutation of *WFS1* gene leading to increase ER stress and cell apoptosis is associated an autosomal dominant form of Wolfram syndrome type 1 Yingying Gong, Li Xiong, Xiujun Li, Lei Su, Haipeng Xiao

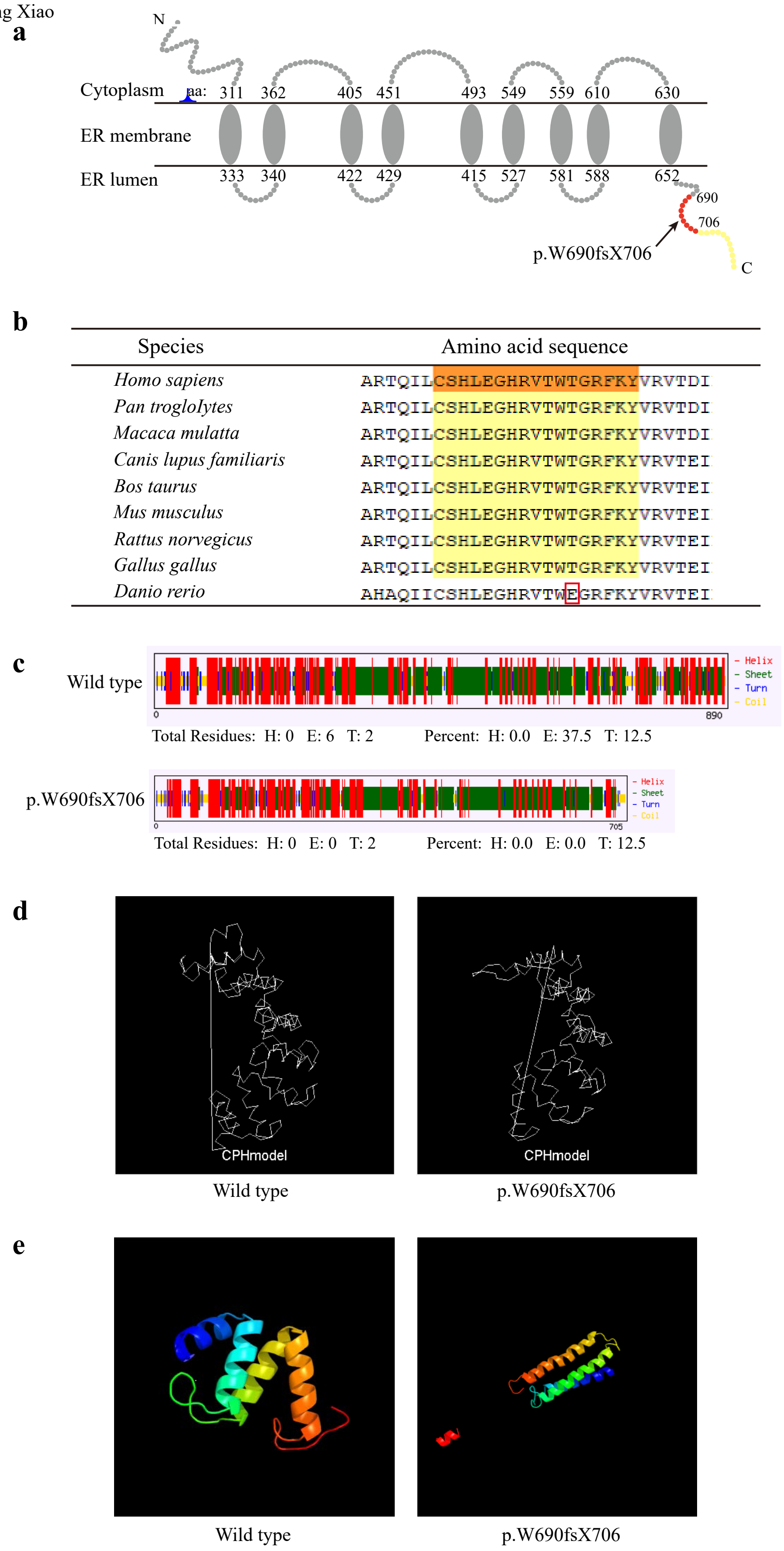

Supplementary Fig. S1 Pathogenicity analysis of p.W690fsX706 mutation. (A) Uniprot database predicted the location of p.W690fsX706 mutation site on wolframin protein structure. The mutant part of amino acids 690-706 was marked with red color, and the missing amino acids were marked with light yellow color. (B) Conservation analysis of the 690-706 amino acid sequence of wolframin protein in various species indicated that this amino acid sequence was highly conserved among mammals. (C) Secondary structure of wolframin protein predicted by CFSSP database suggested that the protein length of mutant (p.W690fsX706) WFS1 was significantly shorter than wild type wolframin, and the frame-shift mutation resulted in 6 fewer β-sheets and different positions for hydrogen bond transitions. H: alpha helix; E: extended strand, participates in beta ladder; T: hydrogen bonded turn. (D) The three-dimensional structure of wolframin protein predicted by CPHmodels software. (E) The three-dimensional structure of wolframin protein predicted by Phyre2 software.
