# Supplementary material for: A novel mutation of WFS1 gene leading to increase ER stress and cell apoptosis is associated an autosomal dominant form of Wolfram syndrome type 1
Source: BMC Endocr Disord. 2021 Apr 21;21:76. doi: 10.1186/s12902-021-00748-z (PMC8059287; doi:10.1186/s12902-021-00748-z)
Supplement: Supplementary file 2 — Additional file 2. [file 12902_2021_748_MOESM2_ESM.pdf]

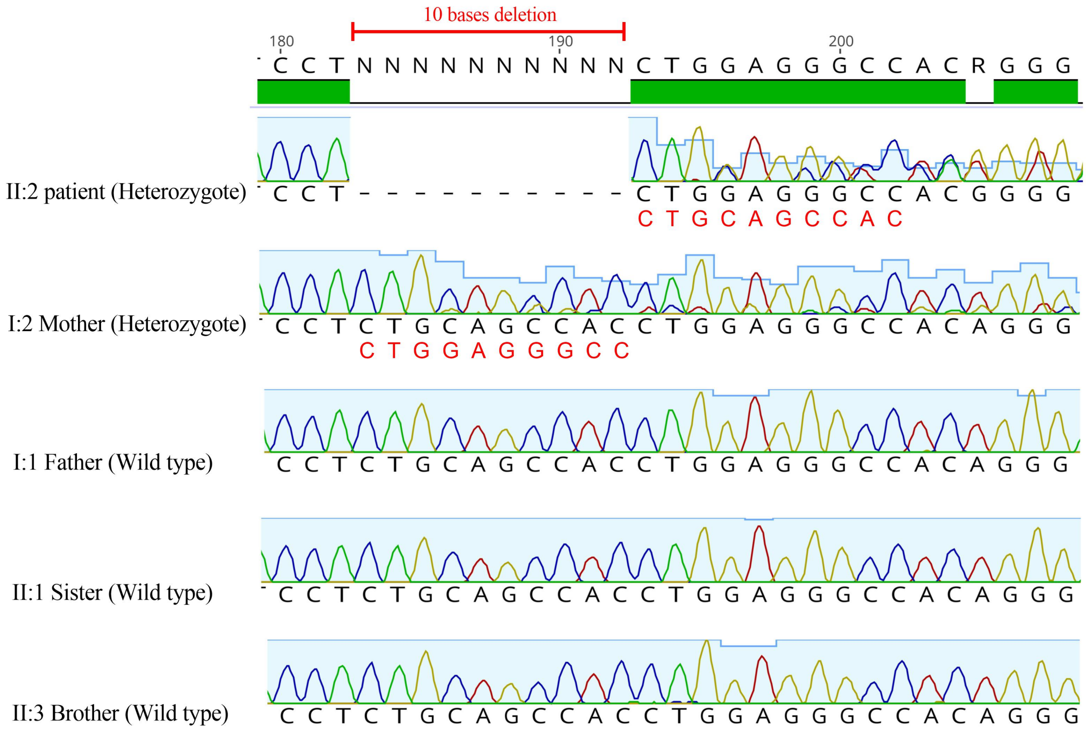

Supplementary Fig. S2 The cDNA sequence chromatograms of *WFS1* exon 8 in the patient and his families. The cDNA sequencing shows heterozygous 10-bp deletion mutation in *WFS1* exon 8 within the mRNA sequence. Horizontal line indicates the deletion site. Double bands appear after the deletion site. The heterozygous 10-bp deletion in exon 8 was also found in the patient's mother except for his father, sister and brother.
