# Supplementary material for: A novel mutation of WFS1 gene leading to increase ER stress and cell apoptosis is associated an autosomal dominant form of Wolfram syndrome type 1
Source: BMC Endocr Disord. 2021 Apr 21;21:76. doi: 10.1186/s12902-021-00748-z (PMC8059287; doi:10.1186/s12902-021-00748-z)
Supplement: Supplementary file 3 — Additional file 3. [file 12902_2021_748_MOESM3_ESM.pdf]

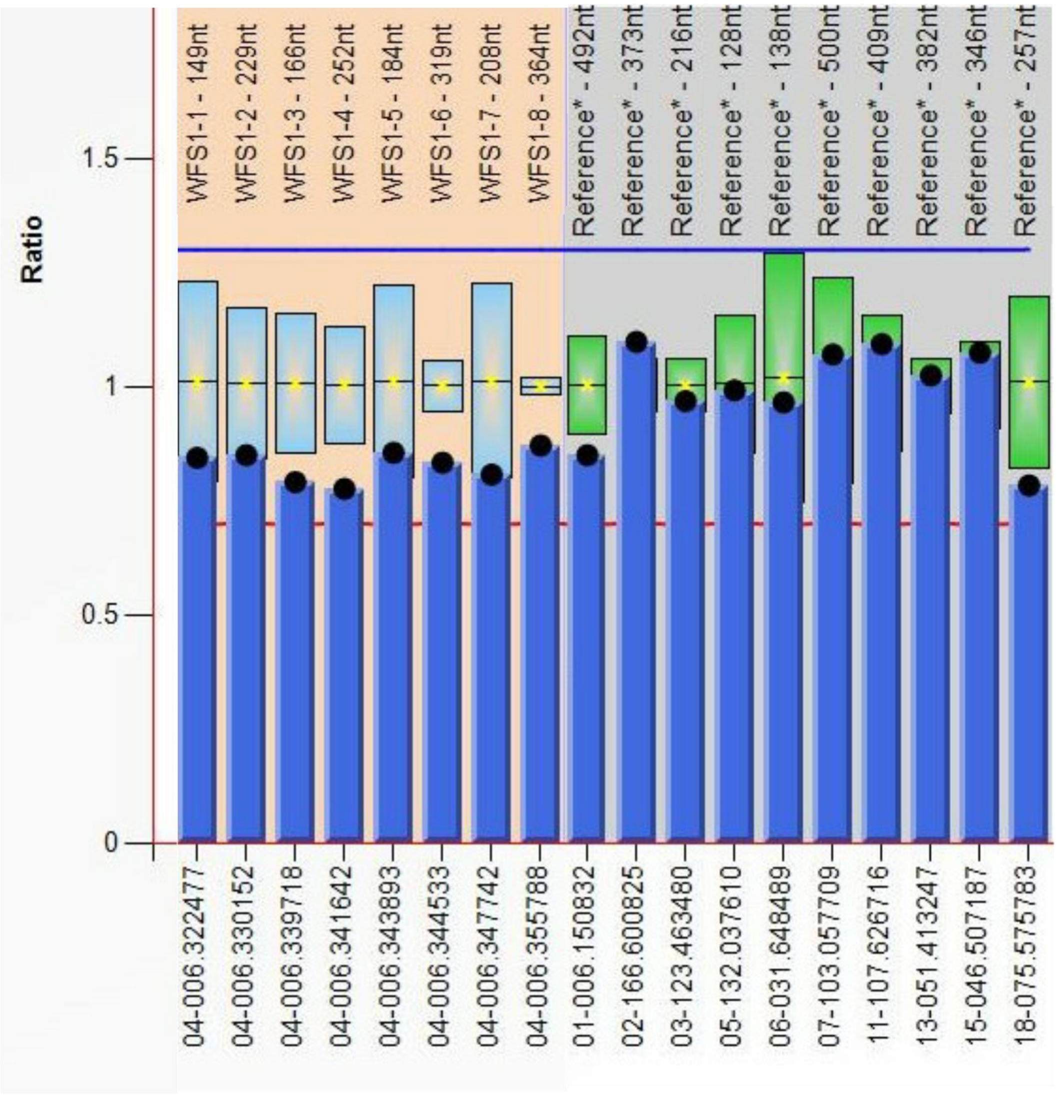

Supplementary Fig. S3 MLPA histogram of the *WFS1* gene. The figure was analyzed by Coffalyser which showed no large deletions/duplication in all the exons of *WFS1* gene in the father.
