# Supplementary material for: A novel mutation of WFS1 gene leading to increase ER stress and cell apoptosis is associated an autosomal dominant form of Wolfram syndrome type 1
Source: BMC Endocr Disord. 2021 Apr 21;21:76. doi: 10.1186/s12902-021-00748-z (PMC8059287; doi:10.1186/s12902-021-00748-z)
Supplement: Supplementary file 4 — Additional file 4. [file 12902_2021_748_MOESM4_ESM.pdf]

A novel mutation of *WFS1* gene leading to increase ER stress and cell apoptosis is associated an autosomal dominant form of Wolfram syndrome type 1

Yingying Gong, Li Xiong, Xiujun Li, Lei Su, Haipeng Xiao

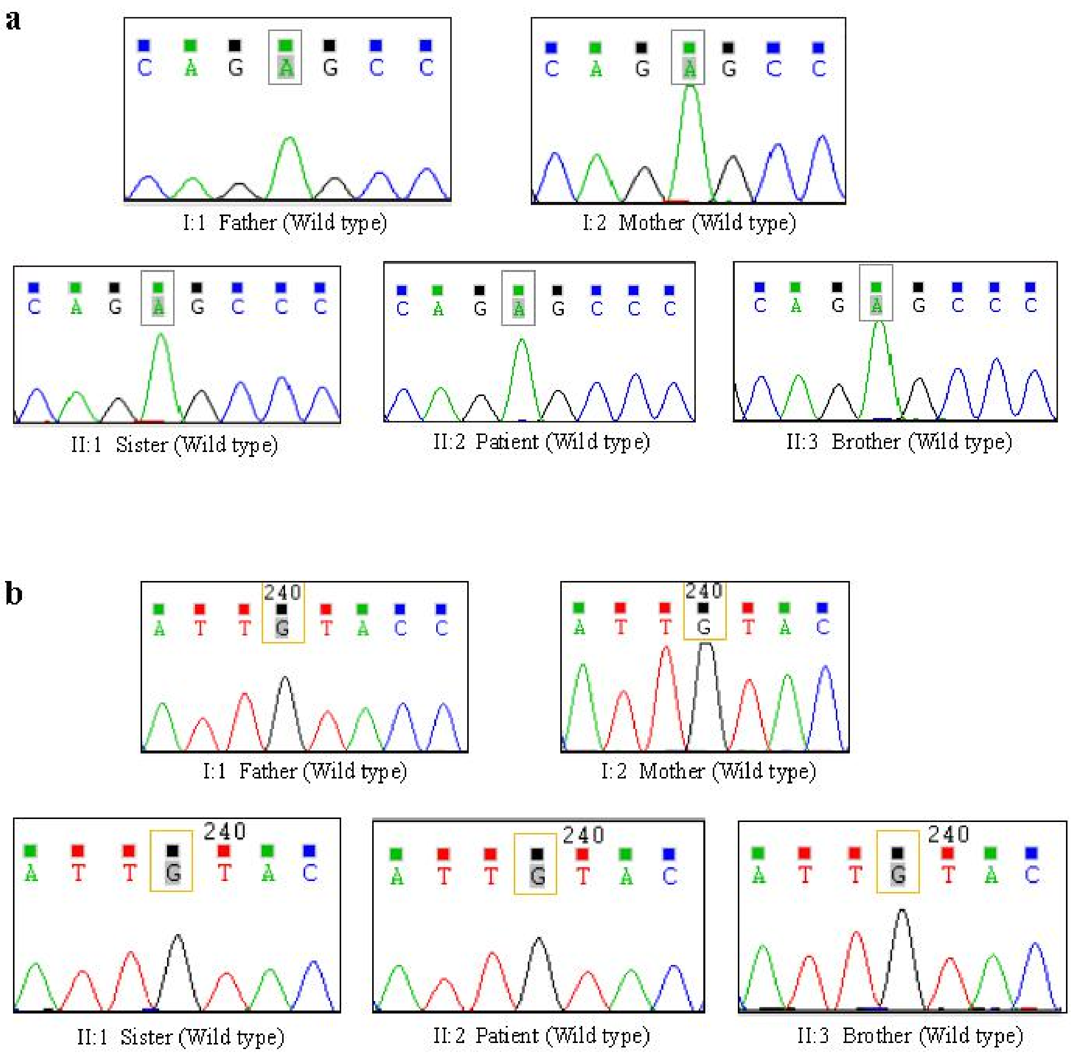

Supplementary Fig. S4 Mitochondrial DNA mutation analysis. (A) None of the patient and his families had mtDNA 3243 A> G mutation. (B) None of the patient and his families had mtDNA 3337 G>A mutation.
